# Critical role of inflammatory cytokines in impairing biochemical processes for learning and memory after surgery in rats

**DOI:** 10.1186/1742-2094-11-93

**Published:** 2014-05-22

**Authors:** Hongying Tan, Jiangbei Cao, Junfeng Zhang, Zhiyi Zuo

**Affiliations:** 1 Department of Anesthesiology, University of Virginia, 1 Hospital Drive, PO Box 800710, Charlottesville, VA 22908-0710, USA; 2 Department of Anesthesiology, Sun Yat-Sen University Cancer Center, 651 Dongfeng East Road, Guangzhou, Guangdong 510060, People’s Republic of China; 3 Department of Anesthesiology, Chinese PLA General Hospital, 28 Fuxin Road, Beijing 100853, People’s Republic of China; 4 Department of Anesthesiology, Shanghai Sixth People’s Hospital, Shanghai Jiaotong University, 600 Yishan Road, Shanghai 200233, People’s Republic of China

**Keywords:** GluR1, Hippocampus, Proinflammatory, Postoperative cognitive dysfunction, Trafficking

## Abstract

**Background:**

Patients with postoperative cognitive dysfunction have poor outcomes. Neuroinflammation may be the underlying pathophysiology for this dysfunction. We determined whether proinflammatory cytokines affect the trafficking of α-amino-3-hydroxy-5-methyl-4-isoxazolepropionic acid receptors to the plasma membrane, a fundamental biochemical process for learning and memory.

**Methods:**

Four-month-old male Fischer 344 rats were subjected to right carotid exposure under isoflurane anesthesia. Some rats received intravenous lidocaine infusion during anesthesia. Rats were tested two weeks later by Barnes maze. The hippocampus was harvested six hours after the surgery for western blotting of interleukin (IL)-1β or IL-6. Hippocampal slices were prepared from control rats or rats subjected to surgery two weeks previously. They were incubated with tetraethylammonium, an agent that can induce long term potentiation, for determining the trafficking of GluR1, an α-amino-3-hydroxy-5-methyl-4-isoxazolepropionic acid receptor subunit.

**Results:**

Surgery or anesthesia increased the time to identify the target box during the Barnes maze test training sessions and one day after the training sessions. Surgery also prolonged the time to identify the target box eight days after the training sessions. Surgery increased IL-1β and IL-6 in the hippocampus. The tetraethylammonium–induced GluR1 phosphorylation and trafficking were abolished in the hippocampal slices of rats after surgery. These surgical effects were partly inhibited by lidocaine. The incubation of control hippocampal slices with IL-1β and IL-6 abolished tetraethylammonium–induced GluR1 trafficking and phosphorylation. Lidocaine minimally affected the effects of IL-1β on GluR1 trafficking.

**Conclusions:**

Our results suggest that surgery increases proinflammatory cytokines that then inhibit GluR1 trafficking, leading to learning and memory impairment.

## Introduction

Millions of Americans receive surgery each year. Postoperative cognitive dysfunction (POCD) has now been recognized to be a significant clinical syndrome because it not only affects the ability of independent living for patients but also increases their morbidity, mortality, and cost of care [1,2]. Thus POCD has a significant impact on patients, their families, and society. Also, POCD can affect patients after cardiac and non-cardiac surgeries, including minor surgeries [2-4]. Advanced age is a significant risk factor for POCD and about 40% or 12% elderly patients (>60 years old) are affected by POCD at hospital discharge or three months post-surgery respectively, after non-cardiac surgeries [2-4]. Thus, it is urgently needed to identify the mechanisms and interventions for preventing or reducing the occurrence of POCD.

It has been shown that neuroinflammation may be the underlying pathophysiology for cognitive dysfunction after general anesthesia or surgery in rodents [5-8]. The role of interleukin (IL)-1β (a proinflammatory cytokine) in this dysfunction has been implicated [5-8]. It has been shown that proinflammatory cytokines can inhibit long term potentiation (an electrophysiological form of learning and memory) and the expression of brain-derived neurotrophic factor (a growth factor contributing to the regulation of learning and memory) in the hippocampus [9-11].

A basic biochemical process for learning and memory is the trafficking of α-amino-3-hydroxy-5-methyl-4-isoxazolepropionic acid receptors (AMPARs) to the plasma membrane in the neurons. This trafficking increases synaptic strength [12-15]. A major mechanism to facilitate this trafficking is via phosphorylation [13,14,16]. For example, phosphorylation of serine 845 in GluR1, an AMPAR subunit, by protein kinase A (PKA), is a well-recognized mechanism for facilitating GluR1 trafficking to the plasma membrane [13].

Based on the above information, we hypothesize that surgery induces proinflammatory cytokine production, which then affects AMPAR trafficking to lead to impairment of learning and memory. To test this hypothesis, we subjected rats to carotid artery exposure, a necessary procedure for carotid endarterectomy in human. We used tetraethylammonium (TEA), which can induce long term potentiation [17,18], to cause GluR1 trafficking in the hippocampal slices. In addition, we tested the effects of lidocaine, a local anesthetic with anti-inflammatory properties [19], on the cognition and cytokine production after the surgery because lidocaine is effective in attenuating the cognitive dysfunction and cytokine production after isoflurane anesthesia [6,19].

## Methods and materials

The animal protocol was approved by the institutional Animal Care and Use Committee of the University of Virginia (Charlottesville, Virginia, United States; Protocol number 3114). All animal experiments were carried out in accordance with the National Institutes of Health Guide for the Care and Use of Laboratory Animals (NIH publications number 80–23) revised in 2011.

### Animal groups

Four-month-old male Fischer 344 rats weighing between 290 and 330 g and obtained from the National Institutes of Health (Bethesda, Maryland, United States) were divided into four groups: control, isoflurane anesthesia, surgery, and surgery plus lidocaine. Rats in the isoflurane anesthesia group was anesthetized by 2% isoflurane for two hours. Rats in the surgery group received right common carotid artery exposure under isoflurane anesthesia (2% isoflurane for two hours). Rats in the surgery plus lidocaine group received the surgery and intravenous lidocaine (1.5 mg/kg as a bolus and then 2 mg/kg/h during the two-hour isoflurane exposure). Since isoflurane was carried by 100% O_2_, rats in the control group were kept in a chamber that was gassed with 100% O_2_ for two hours.

### Surgical procedure and isoflurane anesthesia

As we described previously [20,21], anesthesia was induced by placing rats in a chamber gassed with 3% isoflurane in oxygen. They then were intubated with a 14-gauge catheter and mechanically ventilated with isoflurane carried by 100% O_2_ to maintain end-tidal isoflurane concentration at 2.0%. The inhaled and exhaled gas concentrations were monitored continuously with a Datex™ infrared analyzer (Capnomac, Helsinki, Finland). The usual ventilator settings were as follows: 2 ml as the tidal volume and respiratory rate at 60 breaths/minute. The settings were adjusted to maintain the end-tidal CO_2_ at approximately 32 mm Hg. Rectal temperature was maintained at 37°C ± 0.5°C. Heart rate and SpO_2_ were measured continuously during anesthesia with a MouseOx™ Pulse Oximeter (Harvard Apparatus, Holliston, Massachusetts, United States). At 30 minutes after the onset of isoflurane anesthesia, a 2-cm middle incision was made in the anterior neck after skin preparation and sterilization and subcutaneous tissue infiltration with 0.2 ml of 0.25% bupivacaine. The subcutaneous tissues were dissected bluntly to expose the right common carotid artery. About 1-cm of carotid artery was carefully dissected free from the surrounding tissues. Particular attention was taken to avoid damage to the vagus nerve. The wound was closed with surgical sutures. The whole procedure was performed under aseptic surgical conditions and lasted for 15 minutes. The animals were kept under isoflurane anesthesia for a total two-hour anesthesia. Isoflurane application was then stopped. Rats were extubated and recovered for 20 minutes in a chamber gassed with 100% O_2_ and at 37°C, and were then placed back into their home cage. We anesthetized the rats for 30 minutes before the surgery was started because this length of delay is commonly seen clinically. In addition, surgery and/or anesthesia often lasts two hours or longer in patients. Our previous studies on anesthetic effects on learning and memory involved anesthetizing rats for two hours [19,20], thus we chose anesthesia for two hours for easy comparison.

### Lidocaine application

Lidocaine was infused via a tail vein. As we had done previously [19], lidocaine was dissolved in normal saline to form a solution of 1 mg/ml and given intravenously at 1.5 mg/kg as a bolus and then 2 mg/kg/h during the two-hour isoflurane exposure. The rats in the surgery group received the same volume of saline.

### Barnes maze

Two weeks after surgery, the rats were subjected to a Barnes maze in the same method as we had done previously [19], in order to test their spatial learning and memory. A Barnes maze is a circular platform that has 20 equally spaced holes (SD Instruments, San Diego, California, United States). One hole is connected to a dark chamber that was named the target box. Animals were placed in the middle of the platform each time and were encouraged to quickly find the target box by aversive noise (85 dB) and bright light (200 W) shed on the platform. They first were subjected to a spatial acquisition phase that included training sessions on four days with two trials per day, lasting three minutes per trial and with fifteen minutes between each trial. The spatial reference memory of the rats was tested on days 5 and 12. They were subjected to one trial on each of these two days. The rats were not tested from day 5 to 12. The latency to find the target box during each trial was recorded with the assistance of ANY-Maze video tracking system (SD Instruments, San Diego, California, United States).

### Brain tissue harvest

At six hours after surgery, rats were deeply anesthetized with isoflurane and perfused transcardially with saline. The hippocampi were dissected out immediately for the western blotting of IL-1β and IL-6.

### Western blotting

Brain tissues were homogenized in radioimmunoprecipitation assay buffer (catalogue number: 89900; Thermo Scientific, Worcester, Massachusetts, United States) containing 25 mM Tris–HCl (pH 7.6), 150 mM NaCl, 1% NP-40, 1% sodium deoxycholate, and 0.1% sodium dodecyl sulfate as well as protease inhibitor cocktail (catalogue number: P2714; Sigma, St Louis, Missouri, United States) and protease inhibitor mixture (catalogue number: 1697498; Roche Applied Science, Indianapolis, Indiana, United States). Homogenates were centrifuged at 13,000 g at 4°C for 20 minutes. The supernatant was saved and its protein concentration was determined using the Bradford assay.

Proteins were separated on a polyacrylamide gel and then blotted onto a polyvinylidene difluoride membrane. The membranes were blocked with Protein-Free T20 Blocking Buffer (catalogue number: 37573; Thermo Scientific, Waltham, Massachusetts, United States) and incubated for 16 hours at 4°C with the following primary antibodies: rabbit polyclonal anti-IL-6 antibody (1:500; catalogue number: ab6672; Abcam, Cambridge, Massachusetts, United States), rabbit polyclonal anti-IL-1β antibody (1:1000; catalogue number: ab15077; Abcam, Cambridge, Massachusetts, United States), rabbit polyclonal anti-glyceraldehydes 3-phosphate dehydrogenase (GAPDH) antibody (1:5000; catalogue number: A2228; Sigma, St Louis, Missouri, United States), goat polyclonal anti-GluR1 antibody (1:1000; catalogue number: sc-7609; Santa Cruz, Santa Cruz, California, United States), goat polyclonal anti-phospho-GluR1- (ser845) antibody (1:500; catalogue number: sc-16314; Santa Cruz, Santa Cruz, California, United States), and rabbit monoclonal anti-phospho-PKA C- (thr197) antibody (1:1000; catalogue number: 5661; Cell Signaling, Danvers, Massachusetts, United States). Appropriate secondary antibodies (1:5000) were used. Protein bands were visualized using a Genomic and Proteomic Gel Documentation (Gel Doc) Systems from Syngene (Frederick, Maryland, United States). The target protein band intensities were normalized by the corresponding band intensities of GAPDH from the same samples to reduce loading errors. The results from animals under various experimental conditions then were normalized by the values of the corresponding control animals on the same blot.

### Preparation of hippocampal slices

Two weeks after surgery, rats were anesthetized with isoflurane and then decapitated. Similar to our reported method [22], brains were removed rapidly and placed in ice-cold artificial cerebrospinal fluid (aCSF) bubbled with 5% CO_2_ and 95% O_2_. The aCSF (pH 7.4) contained 116 mM NaCl, 26.2 mM NaHCO_3_, 5.4 mM KCl, 1.8 mM CaCl_2_, 0.9 mM MgCl_2_, 0.9 mM NaH_2_PO_4_, and 5.6 mM glucose. Hippocampal slices at 300 μm thickness were prepared using a vibrating tissue slicer in an ice-cold cutting solution (pH 7.4) containing 260 mM sucrose, 26.2 mM NaHCO_3_, 3 mM KCl, 1.2 mM NaH_2_PO_4_, 5 mM MgCl_2_, and 9 mM glucose, and bubbled with 5% CO_2_ and 95% O_2_. These slices were maintained in aCSF at 4°C for 0.5 hours and then transferred to oxygenated aCSF at 37°C for experiments.

In another experiment, hippocampal slices were prepared from naive four-month old male Fischer 344 rats without surgery or anesthesia. These slices were incubated with IL-1β or IL-6 before TEA stimulation.

### TEA stimulation

Hippocampal slices were incubated for 10 minutes with or without 25 mM TEA at 37°C. This condition is known to induce GluR1 trafficking [17,23].

### IL-1β and IL-6 treatment

Rat recombinant IL-1β (catalogue number: 501-RL/CF) and IL-6 (catalogue number: 506-RL/CF) were obtained from R&D Systems, Minneapolis, Minnesota, United States. Hippocampal slices from naive rats were incubated for one hour with or without 3 ng/ml IL-1β, 3 ng/ml IL-6, 10 μM (2.34 μg/ml) lidocaine, or 10 μM lidocaine plus 3 ng/ml IL-1β in aCSF before TEA stimulation. Those treatment concentrations of cytokines were based on previous studies [24,25]. The lidocaine concentration was chosen because this concentration provided neuroprotection against oxygen-glucose deprivation in hippocampal slices and lipopolysaccharide-induced brain cell injury [26,27].

### Biotinylation of cell surface GluR1 and total cellular protein preparation

Cell surface protein biotinylation was performed essentially as we and others had previously [28-30]. Briefly, hippocampal slices were incubated in aCSF containing 1 mg/ml sulfo-NHS-SS-biotin (sulfosuccinimidyl-2(biotinamido) ethyl-1,3-dithiopropionate; Thermo Scientific, Rockford, Illinois, United States) with gentle shaking during the exposure to TEA or incubation buffer. After incubation, the unreacted reagent was removed by quenching with ice-cold aCSF containing 100 mM glycine twice, for 10 minutes each time. The slices were sonicated and re-suspended in a phosphate buffered saline containing 0.1% (wt/vol) sodium deoxycholate, 1% (vol/vol) Triton X-100, protease inhibitors and phosphatase inhibitors (phosSTOP Phosphatase Inhibitor Cocktail Tablets; Roche, Nutley, New Jersey, United States). Total lysates were centrifuged at 13,000 g for 10 minutes at 4°C to remove nuclei and debris. An aliquot of the lysate (100 μl) was kept aside for western blotting of phospho-PKA, phospho-GluR1, and total GluR1. The remaining sample was incubated overnight with immobilized NeutrAvidin beads (Thermo Scientific, Rockford, Illinois, United States) (300 μl of bead suspension to 150 μl of lysates) in a Pierce centrifuge column (catalog number: 89868, Thermo Scientific, Rockford, Illinois, United States) overnight at 4°C with rotation. The mixture was centrifuged at 1000 g for 3 minutes at 4°C. The beads were then washed three times in phosphate buffered saline containing 1% (vol/vol; Sigma) NP40 and centrifuged at 1000 g for 3 minutes. The bead pellet containing the biotinylated proteins was re-suspended in 200 μl of 2 × Laemmli buffer containing 50 mM dithiothreitol for 30 minutes at 4°C to elute the biotinylated proteins. The biotinylated proteins then were collected in the supernatant after a centrifugation of 1000 g for 5 minutes. The biotinylated proteins were then subjected to western blotting.

### Statistical analysis

Results are presented as means ± SD (n ≥6). The data from the training sessions of the Barnes maze test within the same group were tested by one-way repeated measures analysis of variance followed by the Tukey test. The data from the training sessions of the Barnes maze test between groups were tested by two-way repeated measures analysis of variance followed by the Tukey test. All other data were analyzed by one-way analysis of variance followed by the Tukey test if the data were normally distributed, one-way analysis of variance on ranks followed by the Tukey test if the data were not normally distributed, paired Student’s t test, or Wilcoxon signed-rank Sum test as appropriate. A *P* ≤0.05 was accepted as significant. All statistical analyses were performed with the SigmaStat (Systat Software, Inc., Point Richmond, California, United States).

## Results

No animal died during the surgery or the intended observation period following the surgery. Data for all animals were included for analysis and reported here.

### Surgery impaired learning and memory as well as increased proinflammatory cytokines in the hippocampus

**Figure 1:**
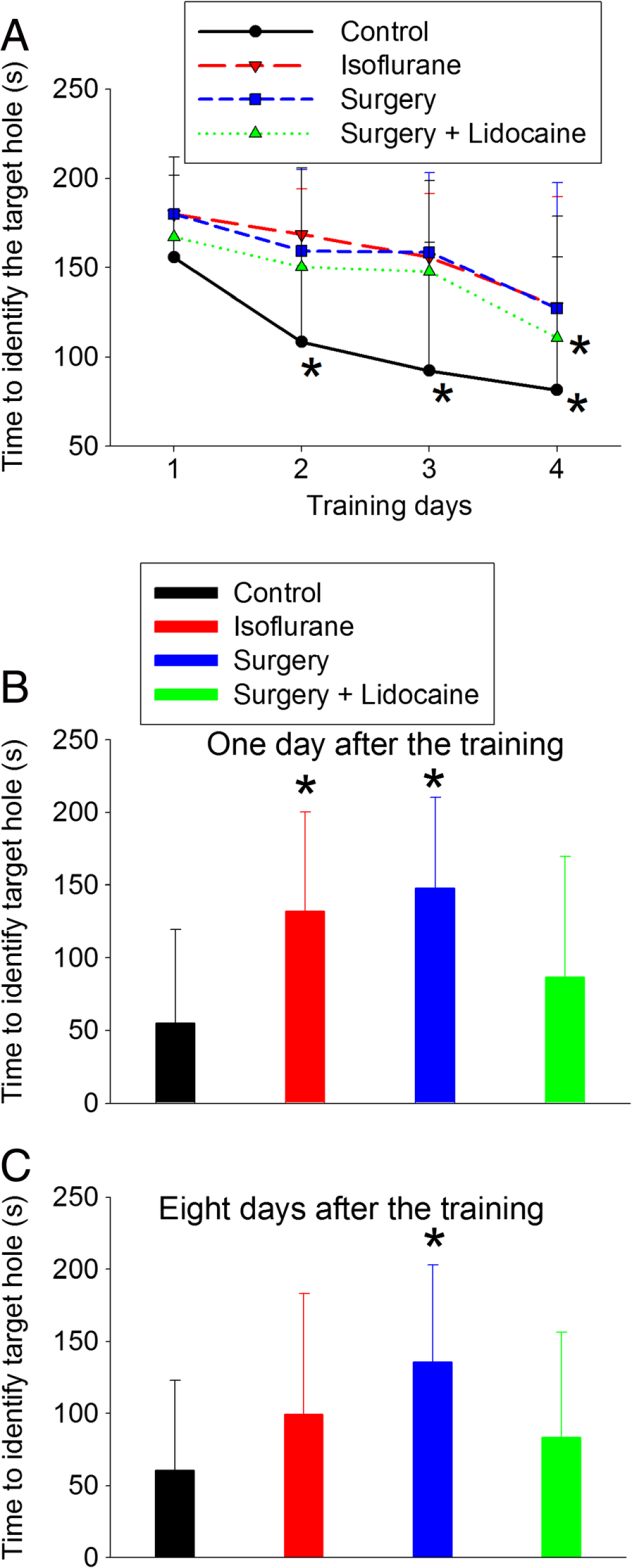
**Performance in the Barnes maze*.*** Four-month-old Fischer 344 rats were subjected to right carotid artery exposure under isoflurane anesthesia with or without intravenous lidocaine. **A**: Barnes maze training sessions started two weeks after the surgery and lasted four days. Results are means ± SD (n = 12-16). *P <0.05 compared with the corresponding data on day one. **B**: reference memory tested at one day after the training sessions. **C**: reference memory tested at eight days after the training sessions. Results are mean ± SD (n = 12-16). *P <0.05 compared with the control group. s, seconds.

The time to find the target box during the four-day training sessions of the Barnes maze test in the control rats decreased with the increase in the number of training sessions. Also, the rats that received surgery plus lidocaine infusion needed a significantly shorter time on day four than that on day one in order to identify the target box. However, this decrease in time with increased training was not apparent in rats after surgery only or anesthesia only. Consistent with this finding, two-way repeated measures analysis of variance showed that surgery or anesthesia had a major effect on the time needed to identify the target box (F (1.29) = 9.53, *P* = 0.004 for surgery; F (1.28) = 11.054, *P* = 0.002 for anesthesia). However, lidocaine was not a significant factor in decreasing the time needed to identify the target box for rats after surgery (F (1.25) = 0.909, *P* = 0.349). When the rats were tested one and eight days after the training sessions, rats in the surgery group took a longer time than control rats to identify the target box (F (3,53) = 5.760, *P* = 0.002 for the data at one day after the training sessions; F (3,53) = 2.963, *P* = 0.040 for the data at eight days after the training sessions). However, the time for rats in the surgery plus lidocaine group was not significantly different from the control rats or rats after surgery only (Figure 1). Rats in the anesthesia only group took longer than control rats to identify the target box at one day after the training sessions. However, this prolongation for the rats subjected to anesthesia only was not apparent at eight days after the training sessions (Figure 1).

**Figure 2:**
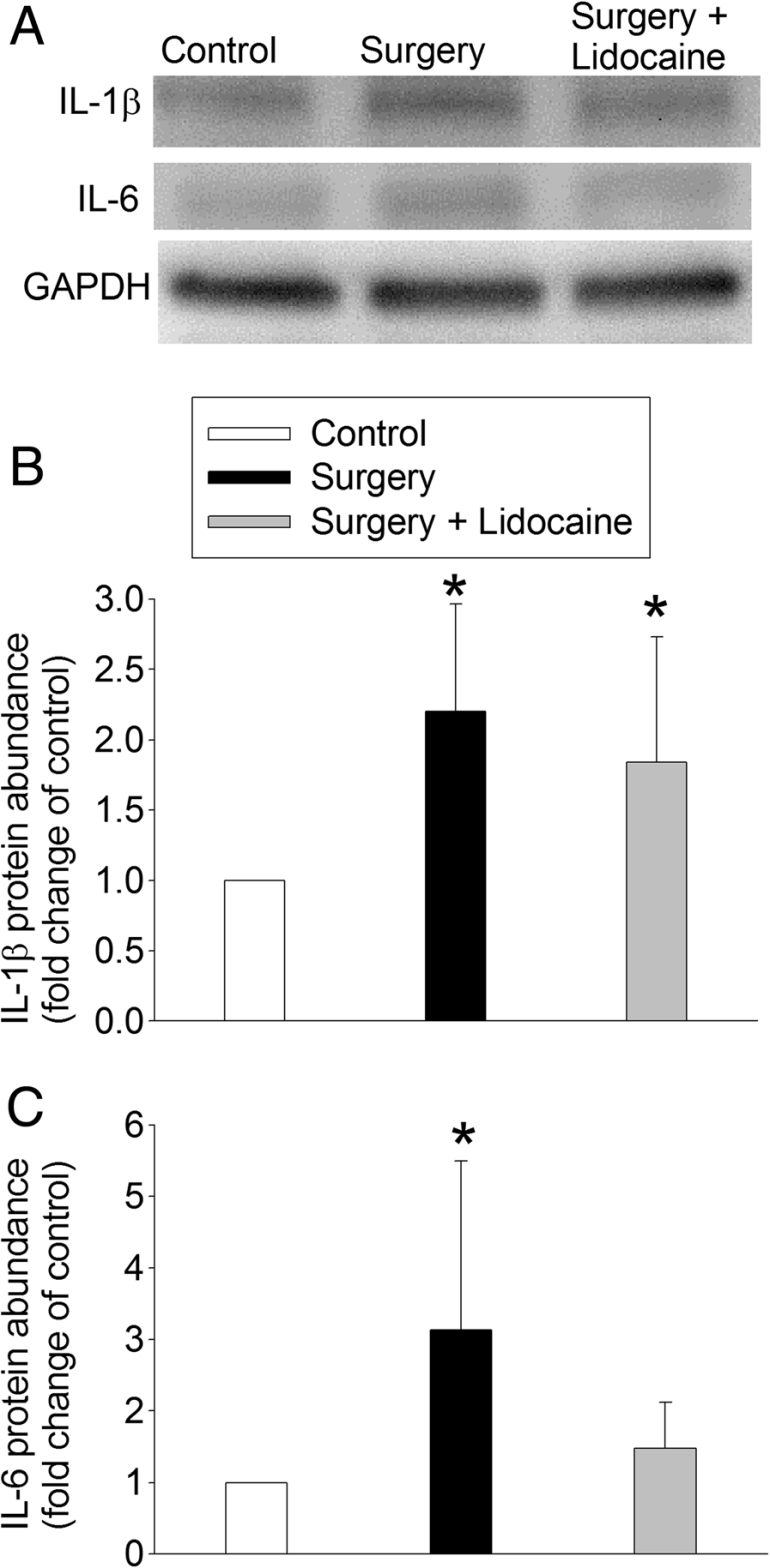
**Surgery-induced IL-1β and IL-6 expression.** Four-month-old Fischer 344 rats were subjected to right carotid artery exposure under isoflurane anesthesia with or without intravenous lidocaine. Hippocampus was harvested at six hours after surgery. **A**: representative western blot images, **B**: graphic presentation of IL-1β results, **C**: graphic presentation IL-6 results. Values in the graphic presentations are expressed as fold changes over the values of control rats and are presented as means ± SD (n = 6). *P <0.05 compared with the control group. GAPDH: glyceraldehydes 3-phosphate dehydrogenase.

The IL-1β level in the hippocampus of rats after surgery or surgery plus lidocaine was significantly higher than that in the control rats (F (2.15) = 5.014, *P* = 0.022). Similarly, surgery also increased the IL-6 level in the hippocampus (F (2.15) = 3.800, *P* = 0.046). The IL-6 level in the hippocampus of rats after surgery plus lidocaine was not different from that of control rats and rats after surgery only (Figure 2).

### Surgery impaired GluR1 trafficking

**Figure 3:**
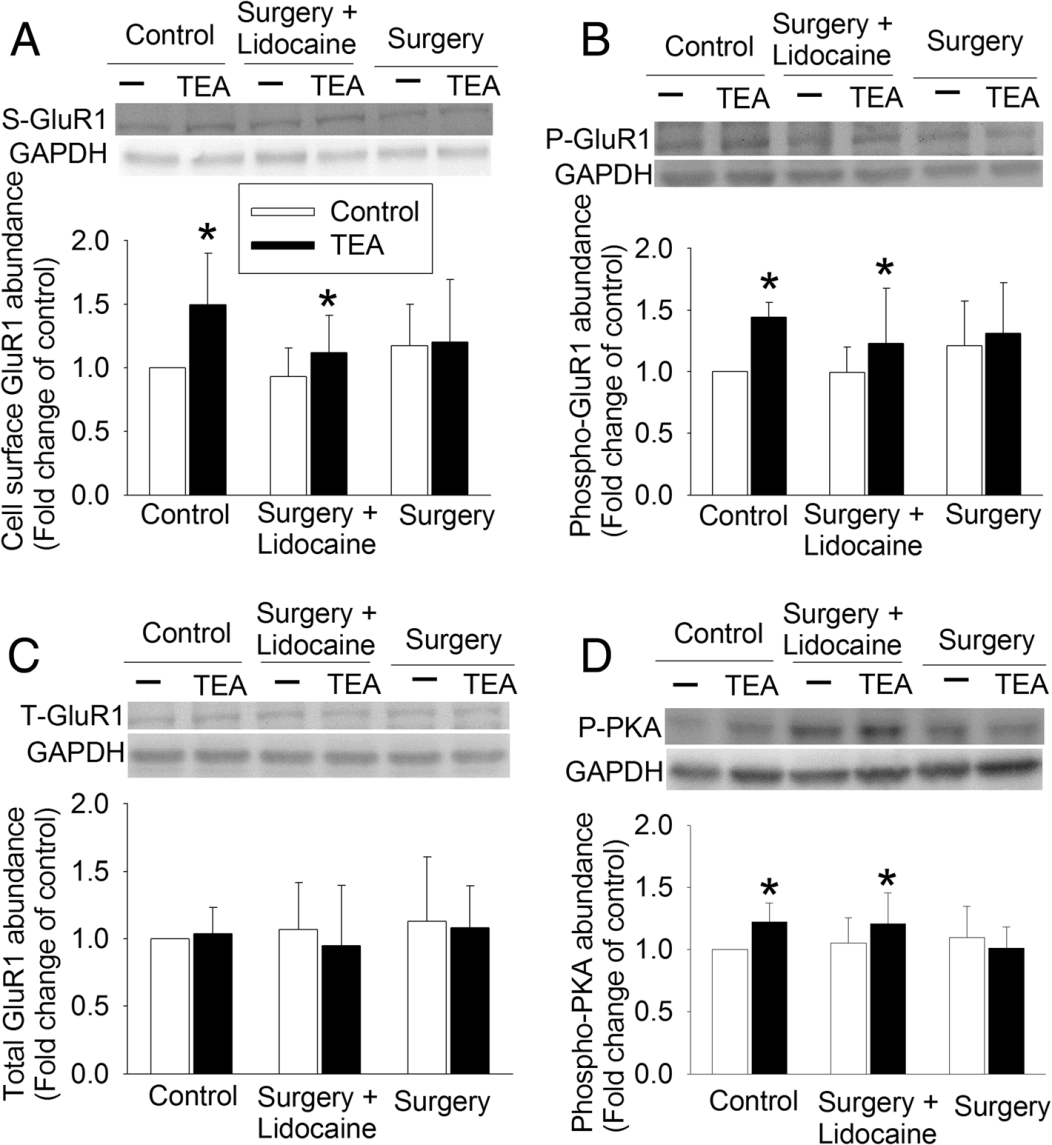
**Inhibition of TEA-induced GluR1 trafficking by surgery.** Four-month-old Fischer 344 rats were subjected to right carotid artery exposure under isoflurane anesthesia with or without intravenous lidocaine. Hippocampus was harvested two weeks after surgery. Freshly prepared 300 μm coronal hippocampal slices were incubated with 25 mM TEA in the presence of 1 mg/ml sulfo-NHS-SS-biotin, a biotinylation reagent, for 10 minutes at 37°C. The homogenates of the hippocampal slices were used for western blotting of phospho-GluR1, phospho-PKA, and total GluR1 and the biotinylated fraction was used for western blotting of GluR1. **A**: biotinylated GluR1, **B**: phospho-GluR1 at ser845, **C**: total GluR1, and **D**: phospho-PKA. Representative western blot images are presented on the top of each panel and a graphic presentation of the results is shown in the bottom of each panel. Results are means ± SD (n = 8-11). *P <0.05 compared with the corresponding control. GAPDH, glyceraldehydes 3-phosphate dehydrogenase; P-GluR1, phospho-GluR1, PKA, protein kinase A; P-PKA, phospho-PKA; S-GluR1, cell surface GluR1; TEA, tetraethylammonium; T-GluR1: total GluR1.

TEA incubation significantly increased GluR1 trafficking to the plasma membrane of the hippocampus from control rats (t (8) = -3.693, *P* = 0.006). Associated with this increased trafficking was the increased phospho-GluR1 at ser845 (t (7) = -10.435, *P* <0.001), a PKA phosphorylation site [13], but did not affect the total amount of GluR1. Consistently, TEA also increased phosphorylated or activated PKA (t (7) = -4.196, *P* = 0.004). A similar pattern of changes occurred in the hippocampus harvested two weeks after the surgery from rats in the surgery plus lidocaine group. However, TEA did not induce GluR1 trafficking and phosphorylation as well as PKA phosphorylation in the hippocampal slices harvested at two weeks after surgery from rats in the surgery group (Figure 3).

### Proinflammatory cytokines impaired GluR1 trafficking

**Figure 4:**
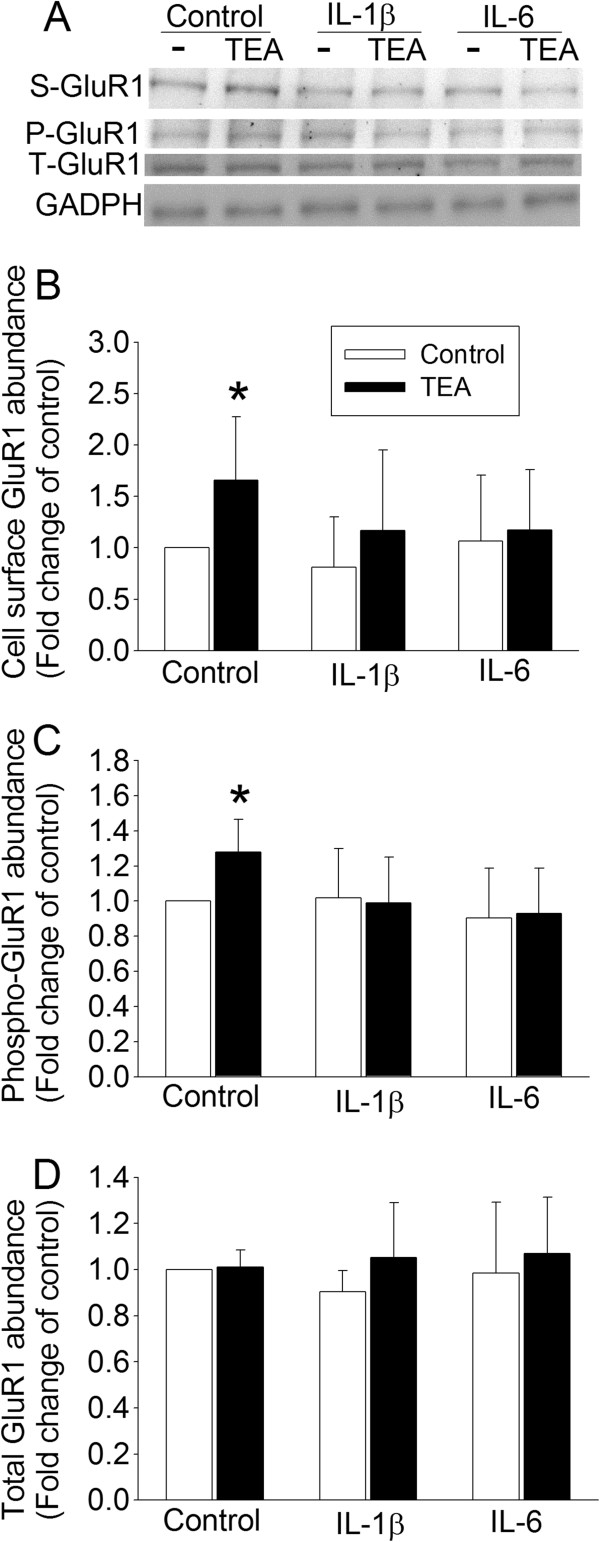
**Inhibition of TEA-induced GluR1 trafficking by IL-1β and IL-6.** The hippocampus was harvested from four-month-old control Fischer 344 rats. Freshly prepared 300 μm coronal hippocampal slices were incubated with or without 3 ng/ml IL-1β or IL-6 for one hour at 37°C and then with 25 mM TEA in the presence of 1 mg/ml sulfo-NHS-SS-biotin, a biotinylation reagent, for 10 minutes at 37°C. The homogenates of the hippocampal slices were used for western blotting of phospho-GluR1 and total GluR1 and the biotinylated fraction was used for western blotting of GluR1. **A**: representative western blot images, **B**: biotinylated GluR1, **C**: phospho-GluR1 at ser845, and **D**: total GluR1. Results are means ± SD (n = 6). *P <0.05 compared with corresponding control. GAPDH, glyceraldehydes 3-phosphate dehydrogenase; P-GluR1, phospho-GluR1; S-GluR1, cell surface GluR1; TEA, tetraethylammonium; T-GluR1, total GluR1.

**Figure 5:**
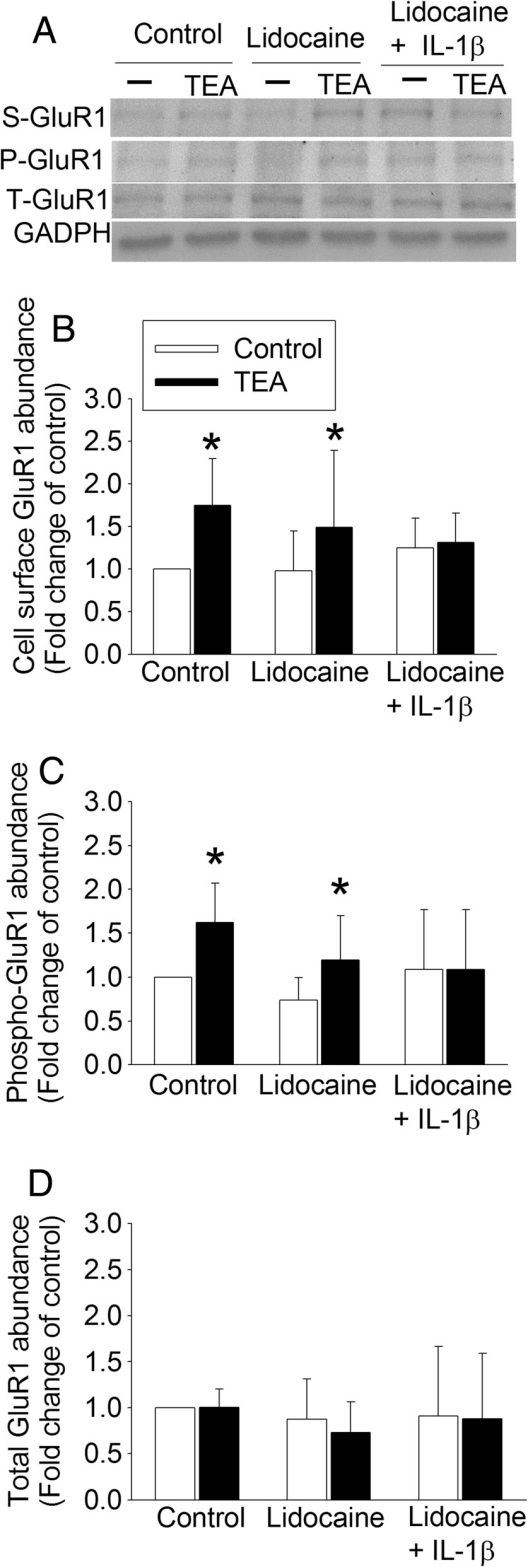
**No effects of lidocaine on IL-1β-induced inhibition of TEA-activated GluR1 trafficking.** The hippocampus was harvested from four-month-old control Fischer 344 rats. Freshly prepared 300 μm coronal hippocampal slices were incubated with or without 10 μM lidocaine, or 10 μM lidocaine plus 3 ng/ml IL-1β for one hour at 37°C and then with 25 mM TEA in the presence of 1 mg/ml sulfo-NHS-SS-biotin, a biotinylation reagent, for 10 minutes at 37°C. The homogenates of the hippocampal slices were used for western blotting of phospho-GluR1 and total GluR1 and the biotinylated fraction was used for western blotting of GluR1. **A**: representative Western blot images, **B**: biotinylated GluR1, **C**: phospho-GluR1 at ser845, and **D**: total GluR1. Results are means ± SD (n = 6). *P <0.05 compared with the corresponding control. GAPDH: glyceraldehydes 3-phosphate dehydrogenase, P-GluR1: phospho-GluR1, S-GluR1: cell surface GluR1, TEA, tetraethylammonium; T-GluR1: total GluR1.

Similar to the above findings, TEA significantly increased GluR1 trafficking to the plasma membrane and phospho-GluR1 but did not affect the total GluR1 in the control hippocampal slices. Incubation of the hippocampal slices with IL-1β or IL-6 abolished these TEA effects (Figure 4). TEA also increased GluR1 trafficking to the plasma membrane (t (5) = −2.576, *P* = 0.050) and the amount of phospho-GluR1 (t (5) = −3.525, *P* = 0.017), but did not affect the total amount of GluR1 in the hippocampal slices incubated with lidocaine. However, hippocampal slices incubated with lidocaine plus IL-1β did not respond to TEA with an increase in GluR1 trafficking and phosphorylation (Figure 5).

## Discussion

Our results clearly showed that surgery under isoflurane anesthesia induced spatial learning and memory impairment because rats after surgery took a longer time to identify the target box of the Barnes maze in the training sessions and the following memory test sessions. We previously showed that intravenous lidocaine infusion significantly attenuated the learning and memory impairment and IL-1β production in the hippocampus of rats after isoflurane anesthesia only [6,19]. However, lidocaine infusion only partly reduced the surgery-induced learning and memory impairment as well as proinflammatory cytokine production in the hippocampus. These findings suggest that the lidocaine regimen that is effective in inhibiting isoflurane effects fails to fully block surgery-induced effects. Part of the reason for this phenomenon may be that surgery under isoflurane anesthesia is a stronger stimulus than isoflurane anesthesia in inducing cognitive impairment and cytokine production. In supporting this possibility, rats after surgery but not rats after anesthesia only took a longer time than control rats to identify the target box at eight days after the training sessions in the Barnes maze test. In addition, the increase of IL-1β in the hippocampus of rats was about 30% after isoflurane anesthesia alone [6] and about 120% after surgery in this study.

Neuroinflammation has been proposed to be the underlying pathophysiology for learning and memory impairment after surgery or anesthesia [5-8]. A recent study showed that enhancing the resolution of inflammation attenuated surgery-induced impairment of learning, memory, and long term potentiation [31]. The role of IL-1β and IL-6 in surgery-induced learning and memory impairment has been indicated [5,6,32]. We have consistently showed that surgery increases IL-1β and IL-6 levels in the hippocampus. Proinflammatory cytokines, such as IL-1β, have been shown to attenuate long term potentiation in the hippocampus [9,10]. It is now well-documented that a fundamental biochemical process for learning and memory is the trafficking of AMPAR to the plasma membrane [12-15]. Thus, affecting AMPAR trafficking may be a possible mechanism for the proinflammatory cytokines to impair learning and memory. Consistent with this possibility, TEA-induced GluR1 trafficking was abolished in the hippocampal slices prepared from rats two weeks after the surgery. In addition, incubation of control hippocampal slices with IL-1β and IL-6 attenuated TEA-induced GluR1 trafficking. This reduced GluR1 trafficking may be due to decreased GluR1 phosphorylation by PKA as surgery also inhibited TEA-induced PKA phosphorylation or activation and GluR1 phosphorylation at ser845, a PKA phosphorylation site [13]. Also, IL-1β and IL-6 inhibited TEA-induced GluR1 phosphorylation at ser845. Thus, our results provide initial evidence on impaired GluR1 trafficking as a mechanism for neuroinflammation and/or proinflammatory cytokines to affect learning and memory functions.

GluR trafficking is a dynamic process. The peak increase of GluR1 trafficking occurs in the hippocampus at around 30 minutes after a fear conditioning stimulus and this increase disappears at two hours after the stimulus [14,33]. Consistent with this known phenomenon, the baseline levels of GluR1 trafficking in the hippocampus were similar among control rats and rats subjected to surgery. TEA induced GluR1 trafficking in the control rat hippocampus but not in the hippocampus of rats after surgery.

Our study showed that lidocaine did not affect TEA-induced GluR1 trafficking and phosphorylation and also failed to attenuate IL-1β-induced inhibition of GluR1 trafficking and phosphorylation caused by TEA. These results suggest that lidocaine does not affect inflammatory cytokines-induced inhibition of GluR1 trafficking. The effects of lidocaine on surgery-induced impairment of GluR1 trafficking and phosphorylation may be due to its influence in the upstream events, such as inhibition of neuroinflammation. Consistent with this possibility, surgery-induced IL-6 in the hippocampus was attenuated by lidocaine. We have shown that lidocaine can inhibit proinflammatory cytokine production from microglial cells stimulated by lipopolysaccharide [27]. Lidocaine can down-regulate nuclear factor-κB signaling to inhibit cytokine production [34]. Thus, the improvement of lidocaine on surgery-induced impairment of learning, memory, and GluR1 trafficking may be due to its anti-inflammatory effects.

Various other mechanisms may play a role in the proinflammatory cytokine-induced impairment of learning and memory. For example, incubation of rat hippocampal neuronal cultures with IL-1β for 24 hours causes synapse loss [24]. IL-1β can also attenuate stimulation-induced brain derived neurotrophic factor production [35]. These structural and biochemical changes may then affect learning and memory functions.

Various surgical procedures have been used in the literature for studying POCD. They include tibial fracture and fixation, hepatic resection and spleen resection [5,36,37]. These procedures are invasive and provide strong surgical stimulation. However, they may significantly affect animals’ immunological functions (such as spleen resection) or motor functions (such as tibial fracture and fixation), which can then affect the evaluation of the animals’ learning and memory assessed by paradigms heavily relaying on motor functions (such as the Morris water maze) during the acute phase after surgery. We performed carotid artery exposure on rats. This procedure is a simple and relatively minor surgery. We performed learning and memory tests two weeks after the surgery to avoid any possible influence of surgical pain on the tests of learning and memory. Our results showed that this surgical stimulation induced learning and memory impairment, suggesting the suitability of this surgical model for POCD research.

Consistent with our previous studies [19,20], isoflurane anesthesia for two hours induced learning and memory impairment. However, surgery may be a stronger stimulus for this effect as rats subjected to surgery needed a longer time than control rats to identify the target box at eight days after the training sessions, while rats exposed to isoflurane anesthesia only did not have this effect. In addition, rats anesthetized by propofol for two hours did not show learning and memory impairment, but rats subjected to carotid artery exposure under propofol-based anesthesia had significant learning and memory impairment in our recent study [38].

Our study has limitations. We focused on determining the biochemical mechanisms for POCD. We used one lidocaine regimen in this study. The failure of this regimen to fully block the surgical effects may be due to an ineffective dosage or the duration of the intravenous lidocaine. Higher lidocaine dosages or longer duration (including during the postoperative period) of lidocaine use may be tested in future studies to determine the usefulness of intravenous lidocaine in reducing POCD.

In summary, we have shown that right carotid artery exposure under isoflurane anesthesia induced learning and memory impairment in adult male rats. This impairment may be due to proinflammatory cytokine-induced interruption of GluR1 trafficking to the plasma membrane, a fundamental biochemical process for learning and memory.

## Abbreviations

aCSF: Artificial cerebrospinal fluid; AMPARs: α-amino-3-hydroxy-5-methyl-4-isoxazolepropionic acid receptors; GAPDH: Glyceraldehydes 3-phosphate dehydrogenase; IL: Interleukin; PKA: Protein kinase A; POCD: Postoperative cognitive dysfunction; TEA: Tetraethylammonium.

## Competing interests

The authors declare that they have no competing interests.

## Authors’ contributions

HT and ZZ conceived the study. HT and ZZ designed the experiments. HT, JC and JZ performed the experiments. HT and ZZ analyzed the data. HT drafted the Methods and materials section. ZZ wrote the manuscript. All authors read and approved the final manuscript.

## References

[B1] Steinmetz J Christensen KB Lund T Lohse N Rasmussen LS Long-term consequences of postoperative cognitive dysfunction Anesthesiology 2009 110 548 555 10.1097/ALN.0b013e318195b569 19225398

[B2] Monk TG Weldon BC Garvan CW Dede DE van der Aa MT Heilman KM Gravenstein JS Predictors of cognitive dysfunction after major noncardiac surgery Anesthesiology 2008 108 18 30 10.1097/01.anes.0000296071.19434.1e 18156878

[B3] Moller JT Cluitmans P Rasmussen LS Houx P Rasmussen H Canet J Rabbitt P Jolles J Larsen K Hanning CD Langeron O Johnson T Lauven PM Kristensen PA Biedler A van Beem H Fraidakis O Silverstein JH Beneken JE Gravenstein JS Long-term postoperative cognitive dysfunction in the elderly ISPOCD1 study. ISPOCD investigators. International Study of Post-Operative Cognitive Dysfunction Lancet 1998 351 857 861 10.1016/S0140-6736(97)07382-0 9525362

[B4] Newman MF Kirchner JL Phillips-Bute B Gaver V Grocott H Jones RH Mark DB Reves JG Blumenthal JA Longitudinal assessment of neurocognitive function after coronary-artery bypass surgery N Engl J Med 2001 344 395 402 10.1056/NEJM200102083440601 11172175

[B5] Cibelli M Fidalgo AR Terrando N Ma D Monaco C Feldmann M Takata M Lever IJ Nanchahal J Fanselow MS Maze M Role of interleukin-1beta in postoperative cognitive dysfunction Ann Neurol 2010 68 360 368 10.1002/ana.22082 20818791 PMC4836445

[B6] Cao L Li L Lin D Zuo Z Isoflurane induces learning impairment that is mediated by interleukin 1beta in rodents PLoS One 2012 7 e51431 10.1371/journal.pone.0051431 23251531 PMC3520904

[B7] Terrando N Monaco C Ma D Foxwell BM Feldmann M Maze M Tumor necrosis factor-alpha triggers a cytokine cascade yielding postoperative cognitive decline Proc Natl Acad Sci U S A 2010 107 20518 20522 10.1073/pnas.1014557107 21041647 PMC2996666

[B8] Tang JX Mardini F Janik LS Garrity ST Li RQ Bachlani G Eckenhoff RG Eckenhoff MF Modulation of murine Alzheimer pathogenesis and behavior by surgery Ann Surg 2013 257 439 448 10.1097/SLA.0b013e318269d623 22964728 PMC3525732

[B9] Chapman TR Barrientos RM Ahrendsen JT Maier SF Patterson SL Synaptic correlates of increased cognitive vulnerability with aging: peripheral immune challenge and aging interact to disrupt theta-burst late-phase long-term potentiation in hippocampal area CA1 J Neurosci 2010 30 7598 7603 10.1523/JNEUROSCI.5172-09.2010 20519534 PMC2891807

[B10] Cunningham AJ Murray CA O’Neill LA Lynch MA O’Connor JJ Interleukin-1 beta (IL-1 beta) and tumour necrosis factor (TNF) inhibit long-term potentiation in the rat dentate gyrus in vitro Neurosci Lett 1996 203 17 20 10.1016/0304-3940(95)12252-4 8742036

[B11] Cortese GP Barrientos RM Maier SF Patterson SL Aging and a peripheral immune challenge interact to reduce mature brain-derived neurotrophic factor and activation of TrkB, PLCgamma1, and ERK in hippocampal synaptoneurosomes J Neurosci 2011 31 4274 4279 10.1523/JNEUROSCI.5818-10.2011 21411668 PMC3086395

[B12] Miyamoto E Molecular mechanism of neuronal plasticity: induction and maintenance of long-term potentiation in the hippocampus J Pharmacol Sci 2006 100 433 442 10.1254/jphs.CPJ06007X 16799259

[B13] Man HY Sekine-Aizawa Y Huganir RL Regulation of {alpha}-amino-3-hydroxy-5-methyl-4-isoxazolepropionic acid receptor trafficking through PKA phosphorylation of the Glu receptor 1 subunit Proc Natl Acad Sci U S A 2007 104 3579 3584 10.1073/pnas.0611698104 17360685 PMC1805611

[B14] Whitlock JR Heynen AJ Shuler MG Bear MF Learning induces long-term potentiation in the hippocampus Science 2006 313 1093 1097 10.1126/science.1128134 16931756

[B15] Rumpel S LeDoux J Zador A Malinow R Postsynaptic receptor trafficking underlying a form of associative learning Science 2005 308 83 88 10.1126/science.1103944 15746389

[B16] Esteban JA Shi SH Wilson C Nuriya M Huganir RL Malinow R PKA phosphorylation of AMPA receptor subunits controls synaptic trafficking underlying plasticity Nat Neurosci 2003 6 136 143 10.1038/nn997 12536214

[B17] Gu J Lee CW Fan Y Komlos D Tang X Sun C Yu K Hartzell HC Chen G Bamburg JR Zheng JQ ADF/cofilin-mediated actin dynamics regulate AMPA receptor trafficking during synaptic plasticity Nat Neurosci 2010 13 1208 1215 10.1038/nn.2634 20835250 PMC2947576

[B18] Suzuki E Okada T TEA-induced long-term potentiation at hippocampal mossy fiber-CA3 synapses: characteristics of its induction and expression Brain Res 2009 1247 21 27 18977337 10.1016/j.brainres.2008.09.101

[B19] Lin D Cao L Wang Z Li J Washington JM Zuo Z Lidocaine attenuates cognitive impairment after isoflurane anesthesia in old rats Behav Brain Res 2012 228 319 327 10.1016/j.bbr.2011.12.010 22192381 PMC3268839

[B20] Lin D Zuo Z Isoflurane induces hippocampal cell injury and cognitive impairments in adult rats Neuropharmacology 2011 61 1354 1359 10.1016/j.neuropharm.2011.08.011 21864548 PMC3189329

[B21] Lee JJ Li L Jung H-H Zuo Z Postconditioning with isoflurane reduced ischemia-induced brain injury in rats Anesthesiology 2008 108 1055 1062 10.1097/ALN.0b013e3181730257 18497606 PMC2666347

[B22] Jung HH Lee JJ Washington JM Zuo Z Inability of volatile anesthetics to inhibit oxygen-glucose deprivation-induced glutamate release via glutamate transporters and anion channels in rat corticostriatal slices Brain Res 2008 1227 234 239 18619419 10.1016/j.brainres.2008.06.063 PMC2617721

[B23] Aniksztejn L Ben-Ari Y Novel form of long-term potentiation produced by a K + channel blocker in the hippocampus Nature 1991 349 67 69 10.1038/349067a0 1845914

[B24] Mishra A Kim HJ Shin AH Thayer SA Synapse loss induced by interleukin-1beta requires pre- and post-synaptic mechanisms J Neuroimmune Pharmacol 2012 7 571 578 10.1007/s11481-012-9342-7 22311599 PMC3415563

[B25] Garcia-Oscos F Salgado H Hall S Thomas F Farmer GE Bermeo J Galindo LC Ramirez RD D’Mello S Rose-John S Atzori M The stress-induced cytokine interleukin-6 decreases the inhibition/excitation ratio in the rat temporal cortex via trans-signaling Biol Psychiatry 2012 71 574 582 10.1016/j.biopsych.2011.11.018 22196984 PMC4732871

[B26] Niiyama S Tanaka E Tsuji S Murai Y Satani M Sakamoto H Takahashi K Kuroiwa M Yamada A Noguchi M Higashi H Neuroprotective mechanisms of lidocaine against in vitro ischemic insult of the rat hippocampal CA1 pyramidal neurons Neurosci Res 2005 53 271 278 10.1016/j.neures.2005.07.004 16102862

[B27] Jeong HJ Lin D Li L Zuo Z Delayed treatment with lidocaine reduces mouse microglial cell injury and cytokine production after stimulation with lipopolysaccharide and interferon gamma Anesth Analg 2012 114 856 861 10.1213/ANE.0b013e3182460ab5 22253275 PMC3310294

[B28] Huang Y Zuo Z Isoflurane induces a protein kinase C alpha-dependent increase in cell surface protein level and activity of glutamate transporter type 3 Mol Pharmacol 2005 67 1522 1533 10.1124/mol.104.007443 15709112

[B29] Huang Y Feng X Sando JJ Zuo Z Critical role of serine 465 in isoflurane-induced increase of cell-surface redistribution and activity of glutamate transporter type 3 J Biol Chem 2006 281 38133 38138 10.1074/jbc.M603885200 17062570

[B30] Rao-Ruiz P Rotaru DC van der Loo RJ Mansvelder HD Stiedl O Smit AB Spijker S Retrieval-specific endocytosis of GluA2-AMPARs underlies adaptive reconsolidation of contextual fear Nat Neurosci 2011 14 1302 1308 10.1038/nn.2907 21909089

[B31] Terrando N Gomez-Galan M Yang T Carlstrom M Gustavsson D Harding RE Lindskog M Eriksson LI Aspirin-triggered resolvin D1 prevents surgery-induced cognitive decline FASEB J 2013 27 3564 3571 10.1096/fj.13-230276 23709617

[B32] Shen X Dong Y Xu Z Wang H Miao C Soriano SG Sun D Baxter MG Zhang Y Xie Z Selective anesthesia-induced neuroinflammation in developing mouse brain and cognitive impairment Anesthesiology 2013 118 502 515 10.1097/ALN.0b013e3182834d77 23314110 PMC3580002

[B33] Wang Z Park S-H Zhao H Peng S Zuo Z A critical role of glutamate transporter type 3 in the learning and memory of mice Neurobiol Learn Mem 2014 doi:10.1016/j.nlm.2014.04.012. [Epub ahead of print] 10.1016/j.nlm.2014.04.012 PMC4143425 24818563

[B34] Lahat A Ben-Horin S Lang A Fudim E Picard O Chowers Y Lidocaine down-regulates nuclear factor-kappaB signalling and inhibits cytokine production and T cell proliferation Clin Exp Immunol 2008 152 320 327 10.1111/j.1365-2249.2008.03636.x 18355353 PMC2384107

[B35] Gonzalez P Machado I Vilcaes A Caruso C Roth GA Schioth H Lasaga M Scimonelli T Molecular mechanisms involved in interleukin 1-beta (IL-1beta)-induced memory impairment. Modulation by alpha-melanocyte-stimulating hormone (alpha-MSH) Brain Behav Immun 2013 34 141 150 23968970 10.1016/j.bbi.2013.08.007

[B36] Wan Y Xu J Ma D Zeng Y Cibelli M Maze M Postoperative impairment of cognitive function in rats: a possible role for cytokine-mediated inflammation in the hippocampus Anesthesiology 2007 106 436 443 10.1097/00000542-200703000-00007 17325501

[B37] Wan Y Xu J Meng F Bao Y Ge Y Lobo N Vizcaychipi MP Zhang D Gentleman SM Maze M Ma D Cognitive decline following major surgery is associated with gliosis, beta-amyloid accumulation, and tau phosphorylation in old mice Crit Care Med 2010 38 2190 2198 10.1097/CCM.0b013e3181f17bcb 20711073

[B38] Zhang J Jiang W Zuo Z Pyrrolidine dithiocarbamate attenuates surgery-induced neuroinflammation and cognitive dysfunction possibly via inhibition of nuclear factor kappaB Neurosci 2014 261 1 10 10.1016/j.neuroscience.2013.12.034 PMC3950371 24365462

